# Prion Disease and the Innate Immune System

**DOI:** 10.3390/v4123389

**Published:** 2012-11-28

**Authors:** Barry M. Bradford, Neil A. Mabbott

**Affiliations:** The Roslin Institute and Royal (Dick) School of Veterinary Studies, The University of Edinburgh, Easter Bush Campus, Midlothian, EH25 9RG, UK; Email: barry.bradford@roslin.ed.ac.uk

**Keywords:** prion disease pathogenesis, transmissible spongiform encephalopathy, innate immune system

## Abstract

Prion diseases or transmissible spongiform encephalopathies are a unique category of infectious protein-misfolding neurodegenerative disorders. Hypothesized to be caused by misfolding of the cellular prion protein these disorders possess an infectious quality that thrives in immune-competent hosts. While much has been discovered about the routing and critical components involved in the peripheral pathogenesis of these agents there are still many aspects to be discovered. Research into this area has been extensive as it represents a major target for therapeutic intervention within this group of diseases. The main focus of pathological damage in these diseases occurs within the central nervous system. Cells of the innate immune system have been proven to be critical players in the initial pathogenesis of prion disease, and may have a role in the pathological progression of disease. Understanding how prions interact with the host innate immune system may provide us with natural pathways and mechanisms to combat these diseases prior to their neuroinvasive stage. We present here a review of the current knowledge regarding the role of the innate immune system in prion pathogenesis.

## 1. Introduction

### 1.1. Prion Diseases

Prion diseases or transmissible spongiform encephalopathies (TSEs) are infectious neurodegenerative conditions characterized by vacuolar degeneration of the central nervous system (CNS) and deposition of an abnormal isoform of the host-encoded prion protein (PrP). These diseases affect a wide variety of animal species and display limited zoonotic potential. Usually displaying prolonged incubation periods, they are clinically recognized via progressive neurological deterioration resulting from synaptic and neuronal loss and associated activated glial responses to CNS damage. Identification of genetically inherited forms of these diseases implicate further the critical role of the prion protein in disease pathogenesis and their classification as protein-misfolding disorders, with similarities to other progressive dementias such as Alzheimer’s and Parkinson’s diseases and amyotrophic lateral sclerosis. Sporadic prion diseases, with no known genetic risk factor or exposure to infection, have also been identified. The disease-associated isoform of the prion protein gains several properties including ability to transmit infection, limited protease resistance, and increased ability to fibrillize and form amyloid, these observations on both etiology and biochemical nature of the agent resulted in the prion hypothesis [1]. 

The prion hypothesis proposed that the infectious agent may be solely composed of a proteinaceous particle, *i.e*., the disease-associated isoform of the prion protein (PrP^Sc^), with the means to self-propagate via an auto-catalytic process of template-mediated refolding of the nascent cellular prion protein (PrP^C^). The pathway and mechanisms from refolding of the prion protein to neurodegeneration are still unknown. The peripheral pathogenesis of these diseases have been extensively studied using animal models, as mice are naturally susceptible to both sheep scrapie and bovine spongiform encephalopathy (BSE). Following natural peripheral exposure to prion agents, infection is usually sequestered to lymphoid organs prior to invasion of the nervous system (termed neuroinvasion) [2,3]. In the absence of local draining lymphoid tissue, subsequent circulation to other lymphoid organs and neuroinvasion are ultimately blocked [4,5]. Using transgenic mouse models or bone marrow chimeric mouse models various hypotheses regarding the genes and cells involved in the prion infectious pathway have been proven or refuted. Though often contradictory, results from these studies have revealed that disease-associated PrP is deposited in lymphoid follicles and replicates upon follicular dendritic cells (FDCs). This stage of pathogenesis has been shown to be dependent upon expression of cellular prion protein by FDCs [6,7]. The lymphoid stage of prion pathogenesis is not always obligatory and is dependent upon various factors including: the host, strain of infectious agent, route of infection and infectious dose [8,9,10,11]. Experimental delivery of prion agent directly to the nervous system may facilitate direct neuroinvasion [12,13]. Direct delivery of the prion agent to the central nervous system may bypass peripheral pathogenesis completely resulting in infection in naturally resistant hosts [14,15]. Concurrent lymphoid sequestration of infection likely still occurs after direct CNS infection and may play a role in subsequent pathogenesis [15,16].

Originating from the stromal cell compartment FDC are specialized cells that capture and retain antigen for presentation to cognate B-cells and subsequent generation of specific antibody responses and thus reside within primary B-cell follicles and germinal centers within lymphoid tissues [17,18]. Due to the reliance of FDCs on lymphotoxin signaling from B-cells to maintain FDC homeostasis [19], immune-compromised models lacking mature FDCs reveal deficits in peripheral prion pathogenesis. As such a positive relationship has been firmly established between the immune-competence of the host and ability to support prion pathogenesis. As with most infectious agents, typical pathogenesis occurs via the preferred route often as a compromise between the agent and the host. Prevention or blocking of this route may promote adaption or alternative pathways of pathogenic activity. For example blocking of intestinal Peyer’s patch formation prior to oral prion infection led to pathogenesis via FDC contained within isolated lymphoid follicles [4]. Similar altered pathogenic routes may be induced following splenectomy or sympathectomy [3,20]. Data obtained from prion pathogenesis studies reveal that rarely infection is blocked completely and more often disease is attenuated, revealing alterations to well-definable characteristics such as the disease incubation period or targeting of pathological changes both peripherally and within the host CNS. The difficulties in analyzing such data are compounded by the problems of global host models (*i.e*., gene-knockout) that effect both peripheral and CNS innate or adaptive immune responses. Despite these difficulties several clear messages have been received regarding the role of specific components of the innate immune system, some of which are less inter-dependent than the systems regulating the adaptive immune response, which we review and summarize below.

### 1.2. The Innate Immune System

**Figure 1:**
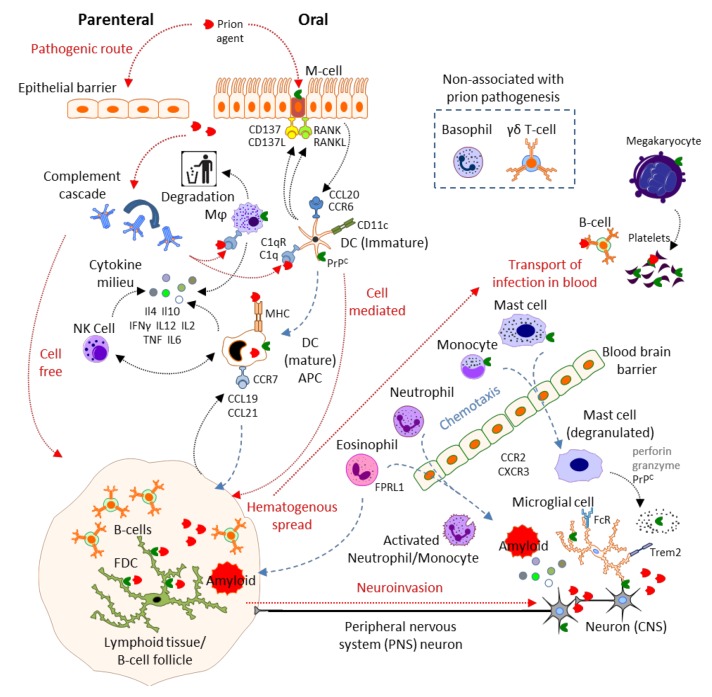
Prion disease and the innate immune system. Adapted with permission from motifolio.com, Biomedical PowerPoint Toolkit Suite.

The innate immune system is considered to be a protective system that is older in evolutionary terms than the adaptive immune system. This system was originally proposed to respond in a non-specific way to invading pathogens in an attempt to eliminate them or their activity, whilst alerting or informing the adaptive immune response. As such the innate immune system confers no immunological memory or lasting protective immunity. The identification and characterization of specific pattern recognition receptors, such as Toll-like (TLR), C-type lectin (CLR), NOD-like (NLR) and RIG-I-like (RLR) receptors, has revealed the diversity of the innate immune system to detect and tailor specific responses to pathogen-associated molecular patterns (PAMPs) [21,22,23,24]. In line with the innate immune system constituting the first line of active defense after physical barrier mechanisms, a significant portion of the cellular component is pre-localized to potential exposure sites. This tissue-resident, or homeostatically maintained, population primarily assesses and responds to insults accordingly, and likely deals effectively with acute insults. A large systemic pool of both cellular (inflammatory) and proteinaceous components of the innate immune system also exists, to be mobilized rapidly in response to signals from epithelia or resident innate immune cells. Below we consider various components of the innate immune system, both cellular and proteinaceous, and their possible roles in prion pathogenesis (summarized in Figure 1).

## 2. Results and Discussion

### 2.1. Physical Barrier to Infection: Epithelial Cells, Microfold Cells

The epithelial cell layers constitute the first physical barrier to infection. Prion infection via the skin is experimentally facilitated by scarification or breaking of the keratinized epidermal barrier layers to allow access to the epithelial layer beneath [25]. Prion infection via the oral or nasal route appears to be naturally more efficient. Prion pathogenesis can be greatly enhanced by disruption of the epithelial layers involved in these routes [26,27]. Under steady state conditions oral uptake of prion infection occurs via specialized microfold cells (M-cells) localized to the follicle-associated epithelium (FAE) of intestinal Peyer’s patches [28,29]. The developmental maturation of M-cells has been shown to require CD137 signaling, with CD137 ligand provided by hematopoietic cells (either B-cells or dendritic cells) [30]. To provide this signal hematopoietic cells are likely required to localize to the sub-epithelial vicinity via chemotaxis mediated by C-C chemokine receptor type 6 (CCR6) responding to C–C chemokine ligand 20 (CCL20), as M-cells do not form if CCR6-CCL20 signaling is prevented [31,32]. The PrP^C^ expressed by M-cells has been hypothesized to function as an uptake receptor for pathogenic bacteria [33]. Translocated PrP^Sc^ has also been observed on enterocyte derived extracellular vesicles suggesting an M-cell independent pathway for prions to cross epithelial barriers [34]. Lumenal sampling by dendritic cells (DC) has been observed [35]. Although this activity by classical DC is rare under steady-state conditions it suggests an additional potential prion uptake mechanism. The de-differentiation of M-cells prior to oral scrapie infection was sufficient to block pathogenesis completely [29], indicating that prion uptake via epithelial and DC mechanisms are insufficient to establish infection in the steady state. The incidence of DC sampling increased following epithelial TLR signaling [36]. Thus the possibility of coincident bacterial infection or other inflammatory stimuli enhancing prion uptake to threshold levels via this mechanism are worthy of further investigation. 

Following uptake and translocation across the epithelial barrier layer, prions are then free to encounter other elements of the innate immune system. Directly beneath the FAE and within basolateral pockets of the M-cells themselves are areas enriched in classical DC, macrophages (Mϕ) and lymphocytes termed the subepithelial dome (SED), precisely located so as to process incoming antigen. The final physical barrier to prion pathogenesis appears to be the distance between site of entry and FDC and subsequently between FDC and peripheral nerves. Innervation of the lymphoid organs has been shown to be critical for prion neuroinvasion [20]. Manipulation to shorten the distance between FDC and peripheral nerves has shown to increase the rate of neuroinvasion [37]. These data provide a strong argument against hematogenous spread of prion agent directly to the CNS. Following aerosol exposure, prion pathogenesis occurs independently of the immune system, FDC, B cells, T cells, NK cells, lymphotoxin β receptor or CD40 ligand signaling [11].

### 2.2. Complement

The complement system is composed of numerous blood borne proteins that are generally synthesized in the liver and circulate as inactive precursors or pro-proteins. Significant amounts of complement components are also synthesized locally by tissue-resident mononuclear phagocytes (MNPs) [38,39], epithelial and stromal cells such as FDC [40]. The complement system is activated by numerous triggers that establish a proteolytic cleavage cascade, amplifying the response and typically resulting in the formation of the membrane attack complex (MAC). The classical complement activation pathway is triggered mainly by antigen-antibody complexes. Alternative activation pathways exist wherein complement component C3b may bind directly to foreign material (alternative pathway) or where mannose-binding lectin recognizes and binds to sugar moieties (lectin pathway) both associated with pathogenic microorganisms. The MAC functions to form transmembrane pores, thereby punching holes in pathogenic cells with the aim of promoting cell lysis and death. 

Complement has been shown to be activated early during prion pathogenesis by as yet undetermined mechanisms and may constitute the first active response to infection. Prion protein has been shown to be directly bound by C1q and Factor H [41] and this binding occurs specifically when prion protein is conformationally modified to represent the conversion to the disease-associated isoform [42]. The role of complement in prion pathogenesis has recently been subject to review [43]. In brief prion or TSE agents are opsonized by complement components including C1q and C3, most likely via the classical complement activation pathway, which may aid in their targeting of the agent to lymphoid follicles. Mice lacking in complement components C1qa, C2 or C3 revealed deficient peripheral prion pathogenesis under specific conditions [44,45]. 

The local production of complement components by MNP has been shown to alter their function [46]. C1q enhances the receptor-mediated uptake of disease-associated PrP by classical dendritic cells (see section 2.4.2 below) [47]. The association of C1q with PrP^Sc^ may alter the downstream processing by classical DC, indeed Flores-Langarica and colleagues imply the receptor calreticulin in this process suggesting targeting towards a protein degradative pathway. This is counter-intuitive to the concept that classical DC may retain intact antigen via a FcγRIIB and complement-mediated process for subsequent presentation [48] which seems more likely to be a requirement for prion pathogenesis under the current prion hypothesis. Uptake of prion agent by other Fc receptors may mediate intracellular processing, e.g., for presentation on MHC class II molecules. Extreme processing of the prion agent is likely to render it ineffective. Cleavage of the cellular prion protein has been shown to protect against infection [49], whilst cleavage of PrP^Sc^ has been shown to modulate prion propagation in a similar fashion [50].

Complement component C1q also regulates dendritic cell development from monocytes via DC-SIGN signaling [51,52]. This induces more tolerogenic DCs and alters their cytokine production, leading to increased Il-10 and reduced Il-12 production and increased phagocytosis of apoptotic cells [53,54]. These data would suggest that C1q deficient mice may also possess a bias in their DC component or functions, as well as a deficiency in immune-complex trapping to FDC, further complicating the interpretation of prion pathogenesis studies performed within them. 

The role of complement during prion pathogenesis is further complicated by the observation that the individual complement components mediating interaction with PrP^Sc^ appear to differ dependent upon the strain of infectious agent [55]. Cell-autonomous and complement-assisted cell-mediated waves of prion trafficking occurs to lymphoid follicles, the relevance of each transport mechanism to disease pathogenesis remains undetermined [56].

The role of the complement system within the CNS has also been extensively reviewed recently [57,58]. As with most innate immune functions within the CNS a clear neuroprotective/neurodegenerative dichotomy exists within the literature, likely indicative that complement activation may lead to both protective and degenerative effects dependent upon the context, responsive and regulatory mechanisms involved. There is currently little evidence supporting a role for complement in prion pathogenesis within the CNS. Mice lacking C1qa, C2 or C3 revealed no deficit to prion pathogenesis following intracerebral inoculation [44] and mice lacking both complement components C3 and C4 revealed unaltered pathogenesis following aerosol exposure to prions [11].

### 2.3. Mast Cells

Mast cells have been implicated in prion pathogenesis due to their high expression levels of cellular prion protein (PrP or PrP^C^) and their ability to traffic to the brain [59]. In fact mast cells are resident, and are also capable of trafficking from the blood to specific regions within the CNS. These different populations of mast cells differentially express the c-kit receptor [60]. Mast cells function to release histamine (via binding of allergens to FcεR1α) and heparin in an early response to infection, and are critical to induce chemotaxis and infiltration of neutrophils and MNPs. Due to the non-inflammatory nature of prion infection there is little evidence to suggest that mast cells undertake this conventional role during peripheral prion pathogenesis. Studies of the activation pathways in mast cells reveal that other factors such as C3a and CCL3 may trigger mast cell degranulation without IgE binding to FcεR1α [61]. This activation pathway is most likely to occur during prion pathogenesis as there is little evidence of the induction of an adaptive immune response and specific anti-prion antibody generation during pathogenesis. The role of immediate local responses to prion infection is in clear need of further investigation to determine the activation effects on mast cells, MNPs and neutrophils amongst other innate immune cell types.

The role of mast cells within the brain is thought to be neuro-modulatory and mast cell trafficking to the brain is linked to steroidal hormones and sexual activity or anxiety behaviors [62]. The presence of mast cells within the brain and their ability to shed expressed PrP upon activation [59] may have implications for prion pathogenesis within the CNS. Shedding of PrP^C^ by mast cells likely occurs via proteolytic or lipolytic cleavage mechanisms removing the glycosylphosphatidylinisotol (GPI) anchor from the protein [59,63]. Activation of mast cells within the CNS may provide copious extracellular PrP^C^ substrate for conversion to the disease-associated isoform late in the disease process, when significant damage has occurred to the CNS already, adding to the exponential accumulation of misfolded protein within the clinical stage of disease. Investigation of GPI-anchorless PrP transgenic mice has revealed that they are capable of forming amyloid in the brain without clinical prion disease symptoms, whilst co-expression of anchorless and native PrP^C^ increased prion pathogenesis [64]. Novel synthetic prion strains have subsequently been generated in the above mentioned co-expressing model [65], suggesting that mast cell involvement during infection may aid in or promote the natural prion strain mutation [66] or prion evolution phenomena [67].

### 2.4. Mononuclear Phagocytes: Microglia, Macrophages, Monocytes, Dendritic Cells and Langerhans Cells

MNP constitute a system of highly phagocytic cells that have a common origin in the bone marrow and circulate or reside in the body tissues in order to sample their environment via uptake of foreign (and self) material. The development of MNP relies upon expression of the colony stimulating factor 1 receptor (CSF-1R) during their differentiation in order to respond to CSF-1. MNP play a vital role in the bridge between the innate and adaptive immune systems by processing and presenting of antigenic compounds to stimulate specific antibody production. During the initial processing by MNP material may be directly disposed of by degradation via lysosomal or proteasomal pathways. These actions of MNP aid in the development of immunity or tolerance to the vast multitude of foreign material encountered. Numerous (particularly intracellular) pathogens have adapted to abuse the functions of MNP as a vehicle for pathogenesis, allowing for their uptake into MNP and trafficking to lymphoid organs for further spread. It is most likely that the uptake and spread of prions to lymphoid organs occurs using MNP as a ‘Trojan horse’ via a similar mechanism.

The role of MNP in prion pathogenesis have been subject to recent review and are considered diverse [68]. This reflects the wide diversity of MNP and their roles in innate immunity. To simplify this diversity MNP can be broadly categorized into the following: (1), resident cells with degradative functions; (2) resident cells with antigen presenting functions and (3) systemic circulating cells responsive to inflammatory stimuli. At present there is little evidence implicating circulating ‘inflammatory monocyte’ populations in prion pathogenesis due to the non-inflammatory nature of infection. 

Regardless of their major functionality all MNP likely use similar surface receptor based pathways and mechanisms to sense their local micro-environment and tailor their responses to pathogens or tissue damage. The exact receptor-mediated mechanism determining the response to prion agents has not been identified or definitively characterized. MNP-mediated uptake of prion agent is enhanced by complement opsonization (see Section 2.1 above). The uptake of prions likely involves complement, lectin or scavenger receptors while there is evidence that Fc [44] (FcγR, RII and RIII deficient mice reveal no deficit following i.c. or i.p. inoculation), or toll-like receptors [69] (at least via the Myd88-dependent pathway) have little role in peripheral pathogenesis under steady state conditions. The downstream processing of the prion agent will likely depend upon the MNP type it has been encountered by.

#### 2.4.1. Degradative MNP

The expression of cellular prion protein in MNP has been associated with phagocytic ability and modulation of inflammatory responses [70,71,72]. Evidence suggests that macrophages (generally identifiable by the markers integrin alpha M, Macrosialin or the F4/80 antigen) degrade the prion agent [73]. The degradative and prion clearance abilities of macrophages appear to be down-regulated when macrophage (Mϕ) activation is stimulated by other danger signal molecules [74]. Again the mechanism of degradation is unknown but represents a critical therapeutic target as it constitutes a natural regulatory mechanism to prion pathogenesis. Stimulating or enhancing this degradative ability may improve peripheral resistance to prion infection. The degradation of agent is countered by the relative replicative ability of the prion agent and its spread through the host. While much has been postulated regarding the prion protein sequence, protein folded conformation and glycosylation in constituting the relative ‘species-barrier’ effect observed relating to limited or lack of cross-species infection ability of specific strains of prion agent (for review see [75]). An alternate hypothesis is to suggest that in prion infection with low or poor replicative ability (*i.e*., due to the factors mentioned above) the balance is switched in favor of degradation of agent, thereby preventing or dramatically slowing pathogenesis. This may occur both within any given cell (and not exclusively to degradative or phagocytic cell types), in fact it is well known that there are limited *in vitro* cell infection models in the prion field due to the relevant inability to infect numerous cell types with a variety of prion strains even within the same species [76], and within the complexity of the host innate immune response.

Cellular degradation of PrP^Sc^ has been shown to be inhibited by cysteine protease inhibitors [77]. It has also been shown that prions may be taken up and trafficked to the endosomal compartment [78] one possible intracellular site of PrP^C^ to PrP^Sc^ conversion [79]. The role of Rab GTPase proteins and their effectors shifting the balance of trafficking away from recycling endosomes and towards late endosomal/lysosomal pathways may play a pivotal role in the cellular decision between propagation and degradation of prions [79]. The cellular ability to uptake and degrade prions appears to be independent of cellular prion protein expression [80,81]. 

#### 2.4.2. Antigen Presenting Cells (APC)

APC have long been identified as being critical to prion pathogenesis. While evidence exists for both cell-free and cell-mediated transport of prions to lymphoid tissues [56], removal of the cell-mediated trafficking severely hampers prion pathogenesis. The basis of cell-mediated trafficking lies in chemotactic efflux of APC to lymph nodes during their maturation [5,82]. The initial phase of transport results from the linked down-regulation of CCR6 and up-regulation of CCR7 allowing APCs to relocate to lymphoid follicles [83]. Prion infection of ‘plt’ mice (deficient in CCL19/CCL21) revealed delayed pathogenesis following transcutaneous infection attributable to impaired CCR7-mediated chemotaxis of DC [82]. Following oral prion infection altered homeostasis in DC levels has been reported in intestinal Peyer’s patches [84]. Transient depletion of CD11c-expressing cells (a commonly used marker indicative of classical DC) revealed the ability to completely block or severely impair pathogenesis via oral and intraperitoneal routes [85,86]. This depletion strategy was observed to eliminate all MNP types from the intestine, including classical DC and macrophages, suggesting that neither transport nor degradation are actively occurring during depletion [87]. Depletion models of CD11c^+^ CD8^+^ (in this case CD8αα) DC subsets restricts intraperitoneal but not oral pathogenesis suggesting that alternative DC subsets may be employed following different infection routes. CD8 knockout mice revealed no alteration of prion pathogenesis, suggesting no direct role for CD8 [88]. This paradigm seems more clearly established in the parenteral ‘skin scarification’ model route of infection. In this model numerous DC and Langerhans cell (LC, expressing the marker Langerin) MNP types are known to interact with the prion agent but the depletion of non-epidermal CD11c^+^ cells had the biggest impact upon pathogenesis [89]. Extracted cell populations representing classical DC and plasmacytoid DC (pDC) have been shown to be capable of transfer of infection *in vivo* [90] and *in vitro* [91] respectively. These findings strongly link these cell types to the retention of intact prion agent and, in the case of classical DC, traffic of the agent in the pre-neuroinvasive stage of prion infection. Resident macrophage cell types within lymphoid organs have so far been shown to have little impact upon disease pathogenesis, indicating that cell free material arriving at the lymphoid tissue is either sequestered to FDC (via complement opsonization) or degraded by resident tingible body macrophages.

#### 2.4.3. MNP in the CNS

Within the CNS the innate immune response is mediated by specialized MNP known as microglia. Microglial development is also dependent upon signaling via the CSF1-R [92], however microglial population of the CNS appears to require both CSF-1R ligands; interleukin-34 (Il-34) [93] and CSF-1 [94]. Microglia activated by amyloidogenic peptides including PrP^106-126^, also reveal enhanced survival and proliferative responses to CSF-1 [95]. Following peripheral prion infection the microglia show signs of activation after neurons and astrocytes have responded. These findings suggest that microglia do not respond directly to presence of misfolded prion protein *per se* but may require priming by other CNS cell types. Following priming the microglial cells undergo chemotaxis to the site of insult [96], impairment of this chemotaxis by knockout of CXCR3 revealed altered central prion pathogenesis [97]. Once activated the microglial population expands and has been shown to upregulate various markers including Trem2, SiglecF, CD200R, and Fcγ receptors in a non-classical immune-response [98]. In fact many of the CNS gene expression profiling studies focused on prion infected CNS have repeatedly identified genes strongly associated with MNP activation (Table 1). Together these data suggest that MNPs constitute the most clinically relevant target innate immune cell population both within and without the CNS during prion pathogenesis. 

**Table 1:** Many of the following genes/proteins have been proven or implicated to have a role in the interaction between prion pathogenesis and the innate immune system and are mentioned in this review.

Gene name	Gene	Uniprot	Phase ^a^	Ref
Allograft inflammatory factor 1	Aif1 / Iba1	O70200	M	[99]
B-cell differentiation antigen CD72	Cd72 / Ly32, Lyb-2	P21855	M	[98]
Beta-2-microglobulin	B2m	P01887	M	[99,100]
Calreticulin	Calr / CRP55	P14211	E	[47]
C3a anaphylatoxin chemotactic receptor	C3ar1	O09047	M	[101]
CAMPATH-1 antigen	Cd52 / Mb7	Q64389	E	[99,100,102]
C–C chemokine receptor type 5	Ccr5 / MIP-1αR, CD195	P51682	E	[96,103]
C–C chemokine receptor type 6	Ccr6 / CD196	O54689	E	[104]
C–C chemokine receptor type 7	Ccr7 / MIP-3βR, CD197	P47774	E	[82]
C–C motif chemokine 2	Ccl2 / MCP-1	P10148	E	[105,106,107]
C–C motif chemokine 3	Ccl3 / MIP-1α	P10855	E	[100,105,106]
C–C motif chemokine 4	Ccl4 / MIP-1β	P14097	E	[106]
C–C motif chemokine 5	Ccl5 / RANTES	P30882	M	[105]
C–C motif chemokine 7	Ccl7 / MCP-3	Q03366	M	[103]
C–C motif chemokine 9	Ccl9 / MRP-2	P51670	M	[99,102]
C–C motif chemokine 12	Ccl12 / MCP-5	Q62401	M	[99,102]
C–C motif chemokine 19	Ccl19 / ELC	O70460	E	[82]
C–C motif chemokine 20	Ccl20 / MIP-3α, Exodus-1	O89093	E	[104]
C–C motif chemokine 21a	Ccl21 / Exodus-2	P84444	E	[82]
C–C motif chemokine 21b		P86792		
C–C motif chemokine 21c		P86793		
CD9 antigen	Cd9	P40240	M	[99,108]
CD40 ligand	Cd40lg / Gp39, CD154	P27548	E	[109,110,111]
CD48 antigen	Cd48 / BLAST-1	P18181	E	[106]
CD209 antigen-like protein A ^b^	Cd209a / DC-SIGN	Q91ZX1		
Cell surface glycoprotein CD200 receptor 4	Cd200r4	Q6XJV4	M	[98]
CMRF35-like molecule 8	Cd300a / MAIR1	Q6SJQ0	M	[98]
Complement C1q subcomponent subunit A	C1qa	P98086	E	[41,44,45,47,100,101,106]
Complement C1q subcomponent subunit B	C1qb	P14106	E	
Complement C1q subcomponent subunit C	C1qc	Q02105	E	
Complement C2	C2	P21180	E	[44]
Complement C3	C3 / HSE-MSF	P01027	M	[44,45,100,101]
Complement C4-B	C4B	P01029	E	[41,99,101,102]
Complement C5	C5	P06684		[112]
Complement C7 ^c^	C7	D3YXF5	E	[113]
Complement component C1q receptor	Cd93 / C1qRp, Ly68	O89103		
Complement receptor type 1	CR1 / CD35	P17927	E	[44,114]
Complement receptor type 2	CR2 / CD21	P19070	E	[44,114,115,116]
C-type lectin domain family 4 member K	Cd207 / Langerin	Q8VBX4		
C-type lectin domain family 7 member A	Clec7a / Dectin1	Q6QLQ4	E	[99,101,102]
C-type lectin domain family 11 member A	Clec11a / Scgf	O88200	L	[108]
CX3C chemokine receptor 1	Cx3cr1	Q9Z0D9	E	[106,117]
C-X-C motif chemokine 9	Cxcl9 / MIG, Scyb9	P18340	M	[118]
C-X-C motif chemokine 10	Cxcl10 / Crg2, Ifi10, Inp10, Scyb10	P17515	E	[100,105,109,113]
C-X-C motif chemokine 11	Cxcl11 / I-TAC, Scyb11	Q9JHH5		
C-X-C motif chemokine 13	Cxcl13 / BLC, Scyb13	O55038	E	[109]
C-X-C chemokine receptor type 3	Cxcr3 / Cmkar3, CD183	O88410	M	[97]
C-X-C chemokine receptor type 4	Cxcr4 / Cmkar4, Lestr, Sdf1r, CD184	P70658	M	[103]
C-X-C chemokine receptor type 5	Cxcr5 / Blr1, Gpcr6, CD185	Q04683	L	[109]
EGF-like module-containing mucin-like hormone receptor-like 1	Emr1 / GpF4/80	Q61549	E	[119]
Eotaxin	Ccl11 / Syca11	P48298		
Forkhead box protein P3	Foxp3	Q99JB6		[120]
Formyl peptide receptor-related sequence 1	Fpr-s1 / Fpr2, Fpr3, Fprl1, Lxa4r	O08790		[121]
Galectin-3	Lgals3 / MAC-2	P16110	E	[106]
Galectin-3-binding protein	Lgals3bp / Cycap, Mama	Q07797	E	[99,101]
Granulins	Grn / PCDGF	P28798	E	[99,117,122]
Granulocyte-macrophage colony-stimulating factor	Csf2 / GM-CSF	P01587	M	[105]
Granzyme B	Gzmb / Ctla1	P04187		
Growth-regulated alpha protein	Cxcl1 / Gro, Gro1, Mgsa, Scyb1	P12850	E	[105]
Guanylate-binding protein 4	Gbp4 / Gbp3	Q61107	M	[99]
H-2 class I histocompatibility antigen, D-37 alpha chain	H2-T23	P06339	M	[99]
H-2 class I histocompatibility antigen, D-D alpha chain	H2-D1	P01900	M	[99]
H-2 class I histocompatibility antigen, K-B alpha chain,	H2-K1	Q7TN03	E	[99]
Hematopoietic progenitor cell antigen CD34	Cd34	Q64314		[123]
High affinity immunoglobulin epsilon receptor subunit alpha	Fcer1a / FcεR1α	P20489		
High affinity immunoglobulin epsilon receptor subunit gamma	Fcer1g / FcεR1γ	P20491	M	[99,101,102,117,122]
High affinity immunoglobulin gamma Fc receptor I	Fcgr1 / FcγRI, CD64	P26151	E	[44,98]
IgG receptor FcRn large subunit p51	Fcgrt / FcRn	Q61559	E	[124]
Integrin alpha-4	Itga4 / CD49d	Q00651	E	[125]
Integrin alpha-X	Itgax / CD11c	Q9QXH4	E	[9,85,90,100,115,126]
Integrin alpha-M	Itgam / CD11b	P05555	E	[96,100,127]
Integrin beta-1	Itgb1 / VLA-4β, CD29	P09055		[128]
Integrin beta-2	Itgb2 / CD18	P11835	E	[101,117]
Integrin beta-7	Itgb7	P26011		[125]
Interferon alpha-inducible protein 27-like protein 2A	Ifi27	Q8R412	E	[99]
Interferon-induced protein with tetratricopeptide repeats 1	Ifit1	Q64282	M	[99]
Interferon-induced protein with tetratricopeptide repeats 3	Ifit3	Q64345	L	[99,108,122]
Interferon-induced transmembrane protein 3	Ifitm3	Q9CQW9	E	[99,108]
Interferon gamma	Ifng / IFNγ	P01580	M	[105,129]
Interferon gamma receptor 1	Ifngr1 / IFNγR1, CD119	P15261		
Interleukin-1 alpha	Il1a / Il1α	P01582	E	[105,106,108,117]
Interleukin-1 beta	Il1b / Il1β	P10749	E	[105,117,118,129]
Interleukin-1 receptor type 1	Il1ra / IL1Rα, CD121a	P13504	M	[118,130]
Interleukin-2	Il2	P04351	E	
Interleukin-2 receptor subunit alpha	Il2ra / Il2Rα, CD25	P01590	E	[120]
Interleukin-4	Il4	P07750	E	[131]
Interleukin-6	Il6	P08505	M	[105,129,132]
Interleukin-10	Il10	P18893	E	[130,131]
Interleukin-12 subunit alpha	Il12p35	P43431		[105,133]
Interleukin-12 subunit beta	IL12p40	P43432	E	
Interleukin 13	Il13	P20109	M	[105,131]
Interleukin 15	Il15	P48346		
Interleukin 18	Il18	P70380		
Interleukin 34	Il34	Q8R1R4		
Killer cell lectin-like receptor subfamily B member 1	Klrb1 / NK1.1, CD161	Q0ZUP1		
Leukocyte-associated immunoglobulin-like receptor 1	Lair1 / CD305	Q8BG84	L	[98]
Leukocyte surface antigen CD47	Cd47 / IAP	Q61735	L	[108]
Leukocyte surface antigen CD53	Cd53	Q61451	M	[99,100]
Low affinity immunoglobulin gamma Fc region receptor II	Fcgr2b / FcγRIIB, CD32	P08101	M	[44,98,99,101,102,117]
Low affinity immunoglobulin gamma Fc region receptor III	Fcgr3 / FcγRIII, CD16	P08508	M	[44,98,99,101,102,117]
L-selectin	Sell / Ly22, LAM1, LECAM1, CD62L	P18337	E	[106]
Lymphocyte antigen 6C1	Ly6c1 / Ly-6C	P0CW02	M	[108,122]
Lymphocyte antigen 75	Ly75 / DEC-205, CD205	Q60767	E	[127,134]
Lymphocyte antigen 86	Ly86 / Md1	O88188	E	[99,102,106,108,122,135]
Lymphotoxin-alpha	Lta / LTα, TNFβ, Tnfsf1	P09225	E	[125,136]
Lymphotoxin-beta	Ltb / LTβ, Tnfc, Tnfsf3	P41155	E	[136]
Lysozyme C-2	Lyz2 / Lyzs	P08905	M	[99,102,137]
Macrophage colony-stimulating factor 1	Csf1 / MCSF	P07141	M	
Macrophage colony-stimulating factor 1 receptor	Csf1r / c-fms, CD115	P09581	M	[117]
Macrophage receptor MARCO	Marco	Q60754	E	[138]
Macrophage scavenger receptor types I and II	Msr1 / Scvr, SRA, CD204	P30204	E	[138]
Macrosialin	Cd68	P31996	E	[99,100,101,106,115,127]
Mannose-binding protein A	Mbl1 / MBP-A	P39039		
Mannose-binding protein C	Mbl2 / MBP-C	P41317		
Mast cell surface glycoprotein Gp49A	Gp49a	Q61450	M	[98]
Mast/stem cell growth factor receptor Kit	Kit / SCFR, c-Kit, CD117	P05532		
MicroRNA 146a	miR-146a		E	[139]
Monocyte differentiation antigen CD14	Cd14	P10810	E	[101,106,115]
Myeloid cell surface antigen CD33	Cd33 / Siglec3	Q63994	M	[98]
Myeloid differentiation primary response protein MyD88	Myd88	P22366		[69]
Perforin	Prf1 / Pfp	P10820		
Probable C-C chemokine receptor type 3	Ccr3/ Cmkbr1l2, Cmkbr3, MIP-1αRl2, CD193	P51678	M	[103]
Prion protein	Prnp / PrP, CD230	P04925	E	[140,141]
Receptor-transporting protein 4	Rtp4 / Ifrg28	Q9ER80	E	[99]
Scavenger receptor cysteine-rich type 1 protein M130	Cd163 / M130	Q2VLH6	E	[113]
Sialic acid-binding Ig-like lectin 5	Siglec5 / SiglecF, CD170	Q920G3	M	[98]
Sialoadhesin	Siglec1 / Sa, Sn, CD169	Q62230	E	[142]
SLAM family member 5	Cd84 / Slamf5	Q18PI6	L	[101]
Sphingosine 1-phosphate receptor 1	S1pr1 / Edg1, CD363	O08530	E	[143]
Stromal cell-derived factor 1	Cxcl12 / SDF-1	P40224	E	[113]
T-cell surface glycoprotein CD3 gamma chain	Cd3g / TCRγ	P11942	M	[116]
T-cell surface glycoprotein CD3 delta chain	Cd3d / TCRδ	P04235	M	[116]
T-cell surface glycoprotein CD4	Cd4	P06332	M	[116,120,144]
T-cell surface glycoprotein CD8 alpha chain	Cd8a / CD8α	P01731	E	[9,116,144,145]
TGF-beta receptor type-2	Tgfbr2 / TGFβR2	Q62312	M	[130]
TNF receptor-associated factor 5	Traf5	P70191	L	[108]
Toll-like receptor 2	Tlr2 / CD282	Q9QUN7	M	[101]
Transforming growth factor beta-1	Tgfb1 / TGFβ1	P04202	M	[108,130,146,147]
Triggering receptor expressed on myeloid cells 2	Trem2	Q99NH8	M	[98,99]
Tumor necrosis factor	Tnfa / TNFα	P06804	E	[106,108,130,131,132]
Tumor necrosis factor ligand superfamily member 9	Tnfsf9 / Cd137l, Cd157l, Ly63l	P41274	E	
Tumor necrosis factor receptor superfamily member 1A	Tnfr1 / CD120a	P25118	E	[130,148,149]
Tumor necrosis factor receptor superfamily member 1B	Tnfr2 / CD120b	P25119	E	[130]
Tumor necrosis factor receptor superfamily member 9	Tnfrsf9 / Ly63, CD137	P20334	E	
Tumor necrosis factor ligand superfamily member 11	Tnfsf11 / RANKL, CD254	O35235	E	[29]
TYRO protein tyrosine kinase-binding protein	Tyrobp / DAP12, CD300d	O54885	E	[98,99,101]
Tyrosine-protein phosphatase non-receptor type substrate 1	Sirpa / Sirpα1, CD172a	P97797	E	[115]

^a^ Reported involvement phase of genes during prion pathogenesis, occurring (E)arly <50%; (M)id 50%–90% or (L)ate >90% during disease incubation period. Data also extrapolated from [150]; ^b^ There are 5 murine homologues (A–E) of human DC-SIGN; ^c^ From MGI:88235 unreviewed Uniprot entry.

### 2.5. Granulocytes: Neutrophils, Basophils and Eosinophils

**Figure 2:**
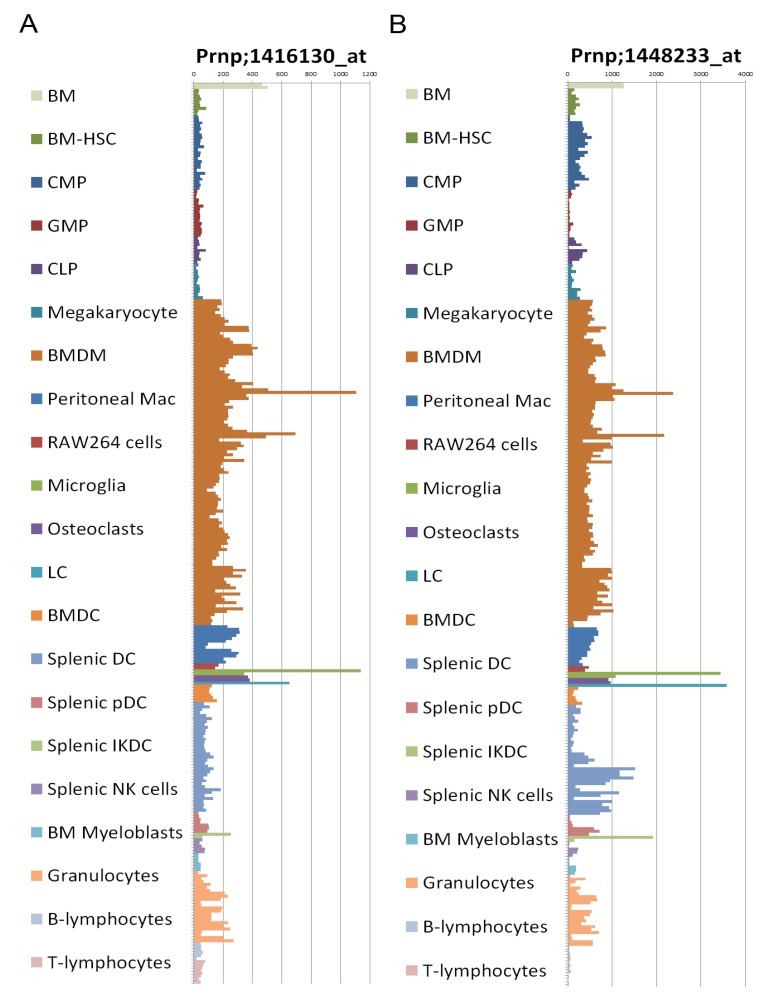
(**a**) *Prnp* expression profiling using Affymetrix GeneChip Mouse Genome 430 2.0 Array probesets 1416130_at and (**b**) 1448233_at; data from [176]. BM, bone marrow; HSC, hematopoietic stem cells; CMP, common myeloid progenitor; GMP, granulocyte-macrophage progenitor; CLP, common lymphoid progenitor; BMDM, bone marrow-derived macrophage; LC, Langerhans cell; BMDC, bone marrow-derived dendritic cell; DC, dendritic cell; pDC, plasmacytoid dendritic cell; IKDC, interferon-producing killer dendritic cell; NK, natural killer.

Gene expression data reveal that PrP expression is generally down-regulated during granulocyte differentiation [151] though some expression is still detectable [152] (see also Figure 2). Neutrophil functions have been shown to be inhibited by both native and scrapie associated prion protein, resulting in failure of neutrophil aggregation and deficits in superoxide radical and beta-glucuronidase export [153]. A 20 amino acid fragment of the prion protein sequence (termed PrP^106^^-126^) has been shown to be directly neurotoxic, activate MNPs and act as a chemotactic agonist for the FPRL1 receptor [121]; the relevance of the PrP^106^^-126^ fragment to prion pathogenesis is elusive at best. FPRL1 is strongly expressed on granulocytes and MNP cell types and promotes chemotaxis to sites in response to allergic inflammation [154]. Eosinophilic inclusions have been observed in both central and peripheral amyloid deposits, although amyloid deposits themselves are eosinophilic, there is some evidence to suggest eosiniophils are present within or around peripheral amyloid deposits. The basis for this localization appears to lie in FPRL1 chemotaxis.

### 2.6. Natural Killer Cells and γδ T Cells

Natural killer (NK) and γδ T cells are thought to play little role in the pathogenesis of prion diseases. Both cell types require triggering or activation by danger signals, for NK cell this is usually via cytokines and for γδ T cells little is known about their activating signals. NK cells primarily respond to virally-infected cells and function to destroy these cells via expression of perforin and granzyme. Perforin-knockout mice revealed unaltered prion pathogenesis [88]. NK cells are typically stimulated via interleukins, 2, 12, 15 and 18 and chemokine CCL5 as well as down-regulation of MHC class I molecules following viral infection. The lack of reported NK cell response to prion pathogenesis suggests that prion agent uptake by monocytes may fail to activate NK cells. There are numerous viral mechanisms used to evade NK cell activation including regulating apoptosis, modulating cytokines and chemokines, and compromising DC functions for review see [155,156,157]. Little evidence has been discovered for any of these functions following prion infection apart from the possible induction of apoptotic cell death mechanisms (in neurons). Recently NK/T cells (a subset of T-cells that express both the NK1.1 marker and αβ T cell receptor) in the spleen have been associated with prion infectivity [91]. Investigating the role of NK/T cells in prion pathogenesis is hampered by the fact that though Rag2^−/−^ mice are deficient in NK/T cells they are unsusceptible to prion infection due to their B-cell deficiency and failure to generate mature FDC. Selective reconstitution of the Rag2^−/−^ model for prion pathogenesis studies has not been reported. An increase in γδ T cells in the peripheral blood mononuclear cells (PBMCs) has been reported following scrapie infection in sheep [116].

### 2.7. Megakaryocytes and Platelets

The megakaryocyte lineage and platelets have been associated with the expression of prion protein [123,158] and MicroRNA miR146a [159]. Following activation of platelets PrP^C^ is released in exosomes [160], similar to the release of exosomal PrP^C^ from macrophages [161]. While not classically considered part of the innate immune system, evidence regarding the expression of TLR2 having a regulatory function has suggested a new role for megakaryocytes in immunity [162]. The over-expression of miR146a, TLR2 and TLR4 has also been reported in microglial cells during scrapie (prion) infection [139]. These data may indicate some common co-expression mechanism of these genes or their possible functional interaction. Regardless of their role in immunity megakaryocytes and platelets constitute a significant portion of the PrP expressing bodies within the blood compartment and as such represent a major vector for prion transmission during blood transfusion [163,164,165,166]. Spread of prion infection between lymphoid organs (*i.e*., from draining to contralateral non-draining lymph nodes) has been prevented by blocking the egress and thus recirculation of B-cells [143]. The routing of prion pathogenesis has been characterized in precise immunohistological detail and describes neuroinvasion from lymphoid organs via the peripheral nervous system [167,168]. Hematogenous spread from lymphoid organs to the CNS has been proposed as an alternative or complementary pathway to neuroinvasion in natural ruminant prion disease with involvement of the circumventricular organs (CVO) in the CNS [169]. 

### 2.8. Prion Protein Expression

Expression of the cellular prion protein (PrP^C^) by the hematopoietic compartment is not required for prion pathogenesis [170], confirming that the innate immune system function in disease pathogenesis operates via non-PrP^C^ dependent mechanisms. Little is known about the uptake mechanisms of prions and the factors that lead to cell clearance, infection or passivity that may facilitate transport. PrP^C^ is not required for uptake of prions *in vitro*, even by non-phagocytic cell types such as neurons [171]. There has been much debate regarding which cells of the immune system do or do not express appreciable levels of PrP. It has long been known that PrP is strongly expressed on hematopoietic stem cells within the bone-marrow compartment; specific lineages appear to lose or down-regulate this expression during differentiation [172,173]. Maturation of cells in response to all-trans retinoic acid is one mechanism known to down-regulate PrP expression [151]. In light of the current debate regarding hematopoietic differentiation and retention or loss of PrP^C^ expression we present here data on the expression of the prion protein gene (*Prnp*) transcripts in various innate immunity relevant cellular compartments (Figure 2). The ability to detect *Prnp* gene expression and cellular prion protein differ, indeed it appears that the mature protein is often more labile or difficult to detect than its corresponding mRNA message, possibly accounting for some of the reported discrepancies [174,175]. The data we present here clearly depicts altered *Prnp* expression levels between subcomponents of the hematopoietic system with Mϕ, DC, Microglia, LC and IFN-producing killer DC displaying the highest observable levels of expressed *Prnp* transcript. 

### 2.9. Transgenic Mice Models

The most common mechanism for determining or proving a correlation between components of the innate immune system (be they cells or proteins) and prion pathogenesis is via transgenic mouse models. These models have been used to determine the effect of knockout or overexpression of a particular protein or cell type on prion pathogenesis. The classic experiment revealed that knockout of *Prnp* resulted in complete resistance to prion infection [140,141]. A plethora of immunity-associated candidate genes have been identified by observational techniques e.g., following gene expression profiling and numerous genes have been screened for a role in prion pathogenesis via knockout transgenic mouse models, e.g., [130] (see also Table 1). The majority of such studies have determined that knockout of an individual component does not prevent prion pathogenesis, for some a slight alteration in pathogenesis was observed and for relatively few a block of pathogenesis was observed and then often only under certain specific conditions. We have performed a pathway analysis of the innate immunity associated genes implicated in prion pathogenesis and determined the major host upstream regulators of prion pathogenesis-associated gene expression (Table 2). 

**Table 2:** Major upstream regulators of genes implicated in prion pathogenesis (Table 1), as determined by Ingenuity pathway analysis IPA (Ingenuity® Systems, www.ingenuity.com).

Upstream regulator	Acronym	P-value of overlap	# of genes
Interleukin-4	Il-4	5.85E-68	73
Interleukin-12	IL-12	4.89E-64	49
Interferon gamma	IFNγ	1.32E-63	80
Interleukin-10	IL-10	3.85E-63	56
Interleukin-2	IL-2	1.05E-56	58
Tumor necrosis factor	TNF	2.70E-53	80
Interleukin-6	IL-6	9.31E-49	57
Colony stimulating factor 2	CSF2	2.85E-46	43

**Figure 3:**
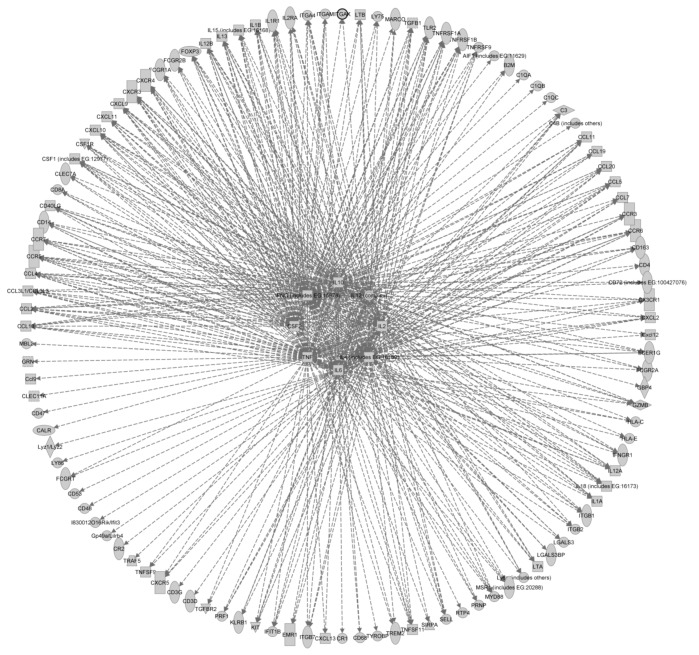
Wheel network diagram showing the overlap of the major upstream regulators of genes implicated in prion pathogenesis. The network was generated through the use of IPA (Ingenuity® Systems, www.ingenuity.com).

From these candidates all have previously been implicated with prion pathogenesis, Il-4, Il-6 and Il-12 have been shown not to be required. Il-10 knockout mice revealed major alterations to, but not prevention of, prion pathogenesis. These data reveal the cytokine milieu occurring during prion pathogenesis and may underlie the basis of alternative activation of MNP and lack of inflammatory, or tolerization of, responses during prion infection. These upstream regulators accounted for 118 out of the 144 genes analyzed presented as a wheel network diagram to reveal the overlap between the regulators and target genes (Figure 3), perhaps revealing why knockout of any individual component may have little overall effect upon prion pathogenesis. Many of these genes and their regulators effect MNP differentiation, maturation and homeostasis as well as modulating MNP responses during infection. These findings indicate that prion pathogenesis is influenced by the steady-state, activation and response of the innate immune system.

## 3. Summary and Conclusions

In summary the innate immune system has many and varied roles in the response to prion pathogenesis, providing both the means to facilitate spread of infection and natural mechanisms to combat infection. The activation (or alternative activation) of mononuclear phagocyte cells appears critical to both peripheral and central prion pathogenesis through as yet unidentified receptors and signaling pathways. Investigation of the expression and function or cellular prion protein has clearly identified widespread expression throughout cell types of the innate immune system and has also suggested possible immune-system specific functions of PrP separate from its possible roles within the CNS.
